# Health assessment of innovative ecological rainforest in pharmaceutical industry: Case study of Zhejiang, China

**DOI:** 10.1371/journal.pone.0281398

**Published:** 2023-02-10

**Authors:** Ying Dong, Ziwei Xiang, Huan Lu, Lei Zhang, Zhiqi Zhao

**Affiliations:** 1 School of Economics and Management, Zhejiang University of Science and Technology, Hangzhou, China; 2 Zhejiang High Quality Opening Research Institute, Hangzhou, China; Adana Alparslan Turkes Science and Technology University: Adana Alparslan Turkes Bilim ve Teknoloji Universitesi, TURKEY

## Abstract

Based on the perspective of ecology and innovation management, this paper selects seven elements from two aspects, innovation subject and innovation environment, to construct the evaluation index system for the health of pharmaceutical innovation ecological rainforest in Zhejiang from 2011 to 2019, together with the entropy weighted TOPSIS method and the obstacle factor diagnosis model. We find that the health of the pharmaceutical industry in Zhejiang can be measured as three stages: stagnation period, recovery period and development period. There is a relative balance between the development of innovation subject and innovation environment. The resilience of innovation subjects, followed by economic and cultural environment, is the key factor hindering the innovation of pharmaceutical industry in Zhejiang. Finally, we propose four countermeasures, including "deploying high-level service chains, broadening investment and financing channels for enterprises, building a reservoir of global talents, and creating an inclusive and open soft environment".

## Introduction

With the deep integration of science and technology innovation, business models and financial capital, the pharmaceutical industry, where life science, information science and pharmaceutical science are intertwined, is gradually transformed from a capital-intensive industry to a technology-intensive industry, deriving new technologies and new business models. However, the pharmaceutical industry is characterized by large investment, long cycle, strong science and technology, multidisciplinary intersection and high return rate, which makes it difficult to achieve innovation by one or a few enterprises alone. Thus, it is necessary to create a stable and dynamic innovation ecosystem.

The tropical rainforest is the most complex and dynamic ecosystem today, which is rich in native carbon, nitrogen and oxygen atoms. These elements create more and more communities, playing various roles in the innovation industry chain, and they build the Silicon Valley innovation ecosystem together in the process of mutual competition. As a "rainforest model", Silicon Valley has been widely studied, and a lot of financial and material resources have been invested to build a "second Silicon Valley", but most of them failed. How to cultivate a "healthy ecological rainforest of innovation" has become the research focus. Creating innovative ecological rainforest is an important way to boost the high-quality development in the pharmaceutical industry and constructing an evaluation system of ecological rainforest health can measure the innovation ability and development level of the pharmaceutical industry. This paper aims to shed light on the following question: First, what is the healthiness of the innovation ecological rainforest? How to assess and judge the healthiness of innovation ecological rainforest? Second, we introduce the innovation ecological rainforest theory to the pharmaceutical industry, but what elements are included in the innovation ecological rainforest of the pharmaceutical industry? How to build a structure model of pharmaceutical innovation ecological rainforest? How to construct the index system for assessing the health of pharmaceutical innovation ecological rainforest? Third, based on the real conditions on the development of the pharmaceutical industry in Zhejiang Province, and the analyses of the development of the innovation ecological rainforest health status of the pharmaceutical industry in Zhejiang Province from 2011 to 2019, what are the obstacle factors to the innovation ecological rainforest of the pharmaceutical industry? Therefore, we apply entropy weighted TOPSIS method and the obstacle factor diagnosis model to evaluate the health level of pharmaceutical industry in Zhejiang, from 2011 to 2019. The entropy TOPSIS method is an improvement of the traditional TOPSIS evaluation method, it calculates the weights of evaluation indicators by the entropy method, and then ranks the evaluation objects by the TOPSIS method through the similarity to the ideal solution. The entropy method is to objectively determine the weight of each evaluation index based on the information provided, while TOPSIS method is to define the distance between the optimal solution and the worst solution of the decision problem, and finally calculates the relative similarity of each solution to the ideal solution to rank the solutions as superior or inferior. Combining these two methods, we can effectively eliminate the influence of subjective factors in determining the weights, and analyse the moving trend of the health of the pharmaceutical innovation ecological rainforest in Zhejiang Province. This paper contributes to the literature by providing its own insights on the health of innovation ecological rainforest and constructing a pharmaceutical innovation ecological rainforest health assessment index system, with empirical research evidence.

The rest of paper proceeds as follows. The next section reviews literatures studying the health assessment of pharmaceutical industry and innovative ecological rainforest. The third section outlines data and model in the evaluation process. The fourth section evaluate the health of pharmaceutical industry in Zhejiang. Final section concludes.

## Literature review

### Basic features of innovative ecological rainforests

Innovative ecological rainforest was firstly introduced by Huang and Hollowett (2012) [1], and they compare the innovation ecosystem of Silicon Valley to a tropical rainforest likened the innovative ecological system in Silicon Valley to a tropical rainforest. Huang (2019) [2] describes this ’rainforest model’ with characteristics of biodiversity accumulation, mutually beneficial symbiosis and rapid flow of innovative elements. The ’rainforest model’ could been presented as an ecosystem formed by non-linear and self-organization dissipation structure, with internal sustainability.

In natural ecosystem, tropical rainforest can be divided into two parts: biotic community and abiotic environment, of which biotic community includes producers, consumers and decomposers, and the abiotic environment refers to the non-living elements in the rainforest, such as sunlight, air, water, etc., which are the necessary conditions for the survival and development of various subjects in the rainforest. Similarly, the innovation and development of the pharmaceutical industry is also inseparable from the subjects and the environment: the innovative subject includes pharmaceutical enterprises, universities, research institutes, governments, financial institutions, intermediary service agencies, users and other individuals and organizations, mainly responsible for original innovation and providing services for early technological innovation. The innovative environment includes four aspects: economy, politics, ecological physics and culture, they provide various types of nutrition required for the development and growth of innovative subjects.

In an ideal innovation rainforest model, any element is free to link and combine with all elements inside and outside the system at will to achieve self-breakthrough. In reality, innovation activities are always restricted by various barriers such as geography, culture, institution, legal, knowledge, and technology, which increase transaction costs. In the rainforest of innovation, there are key species that play the central support role, which generally have the power to integrate and influence, acting as a mediator of social trust, shortening the distance of communication, connecting various scattered organizations, and promoting the valuable interaction of various elements in the rainforest. If key species are lost, the biodiversity of innovative rainforests will be destroyed and many species will not exist any longer.

### Health assessment of innovative ecological rainforests

McMichael et al. (1999) [3] define Ecosystem Health as a discipline that studies the aspects of prevention, diagnosis, and omen of ecosystem management, as well as the relationship between ecosystem health and human health. Li and Zeng (2015) [4] indicate that we can grasp and evaluate the operating state of innovative ecosystem and provide new ideas for their healthy development, through the study of the health of innovative ecosystem. The main methods for evaluate the health of innovative ecosystem are the indicative species method and the indicator system method. Yang et al. (2010) [5] suggest that the indicative species method is to evaluate the health of the system based on changes in the quantity, productivity, structural function, and other elements of the ecosystem’s key species, endemic species and endangered species. The indicator system method refers to building a suitable innovation ecosystem health evaluation system, through classification, collation, and the analysis of their embodied significance and meaning, based on the selection of representative indicators. The indicator system method includes analytic hierarchy method, principal component analysis method, health distance method, VOR comprehensive numerical evaluation method and TOPSIS method.

There is diversity regarding to the selection of indicators for the health assessment of rainforest ecosystem, but the indicator system should contain vitality, structure and resilience, as in Costanza, Norton, and Haskell (1992) [6]. Mezzourh and Nakara (2012) [7] believe that there are complex material and energy exchanges among elements in the ecological rainforest, and that there are many ’cornerstone species’ in the rainforest, as a binder to connect subjects and elements. Tian, Huang, and Xiao (2017) [8] construct three primary indicators, including vegetation structure, ecological services and ecological environment, they use the material-element model and class II survey data to evaluate the health level of forest ecosystem in the Lanling Creek Basin. Zhou, Ye, and Zhao (2021) [9] divide the Altai Mountain into seven woodland areas and evaluate the health status of the Altai Mountain woodland with the VOR composite index model.

On one hand, we can evaluate the health of innovative ecosystem from the perspective of ecology. Yao, Gao, and Zan (2019) [10] construct a health evaluation index system for enterprise innovation ecosystem, including primary indicators of productivity, adaptability and diversity; innovation ecosystem suitability evaluation model in Liu, Zhang, and Deng (2019) [11] is based on four dimensions: openness, synergy, sustainability and growth; Gu and Hu (2020) [12] build a multi-stage health evaluation model with growth, symbiosis, balance and regenerative force as primary indicators.

On the other hand, we can take the perspective of innovation management to evaluate the health of the innovative ecosystem. Durst and Poutanen (2013) [13] measure the success of the innovative ecosystem in terms of resources, culture, and regulatory environment. Zhang, Chen, and Lang (2018) [14] construct an evaluation index system containing indicators such as scale and growth ability, scientific and technological innovation, output capacity, R&D investment, technology acquisition and transformation investment, for the survival and development of high-tech industry.

### Pharmaceutical innovation ecological rainforest health impact factors

First, government policy support is an important factor to enhance the ecological health of pharmaceutical industry innovation. Chen and Shi (2013) [15] use SAS system to study the relationship between China’s pharmaceutical industry policy and pharmaceutical innovation, finding that there is a positive correlation between national policy support and the R&D capability of pharmaceutical enterprises. Li, Wang and Qian (2018) [16] discuss the pharmaceutical industry policy system in the U.S. Although the scale of government R&D support funds in the U.S. is small, its industrial policy objectives are clear and various departments coordinate with each other to form a perfect policy network. Chen, Li and Chen (2020) [17] argue that government regulation helps to promote the healthy development of the pharmaceutical industry, and it improves the scientific regulation of pharmaceutical industry in China.

Second, pharmaceutical enterprises are the main body of innovation activities. Cai (2009) [18] summarize the development routine of the pharmaceutical industry in the U.S. and concluded that research outcomes from academic institutions and external fundings are key factors in promoting the development of the pharmaceutical industry, indicating that the low investment in R&D by Chinese enterprises and the imperfect system of industry-academia-research are the main obstacles to the development of the pharmaceutical industry in China. Hu and Wu (2015) [19] conclude that corporate mergers and acquisitions benefit the performance of pharmaceutical companies. Kang and Zhu (2020) [20] studied the relationship between corporate social responsibility and innovation performance of pharmaceutical companies using the paradigm of "resource-capacity-performance", they find that there is a significant positive relationship between the two.

Innovative ecological rainforest is a special form of innovative ecosystem, and its health assessment should be considered from the perspective of ecology and innovation management. At present, the research on innovative ecological rainforests in China is still in its infancy. Therefore, we introduce the concept of innovative ecological rainforest to the pharmaceutical industry to build a health assessment system and study the innovative development of Zhejiang pharmaceutical industry. The result can help to promote the modernization of drug safety governance system, and boost the high-quality development of the pharmaceutical industry.

## Methodology and data

### Health assessment index system of innovative ecological rainforest in pharmaceutical industry

The index system for health assessment, as shown in Table 1, has two dimensions: innovative subject and innovative environment. In the natural ecosystem, the rainforest can be divided into two parts: biotic community and abiotic environment. By analogising to the pharmaceutical innovation ecological rainforest, the primary indicators are set as innovation subject and innovation environment. The innovation subject corresponds to the producer, consumer and decomposer in the ecological rainforest system, mainly responsible for original innovation and providing services for early technological innovation. The innovation environment is similar to the inorganic environment of sunlight, air and water in nature to support for the development of innovation subjects.

**Table 1:** Health assessment index system for pharmaceutical industry.

Primary indictor	Secondary indicator	Tertiary indicator	Unit
Innovative Subject A	Vitality A1	Sales in Pharmaceutical Industry A11	100 M Yuan
		Profit in Pharmaceutical Industry A12	100 M Yuan
		No. of Patent Application by Pharmaceutical Industry A13	n/a
		Sales of New Product A14	100 M Yuan
	Organizational Structure A2	No. of R&D Institutions A21	n/a
		No. of Higher-Education Institutions A22	n/a
		Year-end Deposits in Financial Institution A23	100 M Yuan
		No. of Law Firm A24	n/a
	Resilience A3	No. of Pharmaceutical Manufacturing Enterprises A31	n/a
		Average No. of Employees A32	n/a
		No. of R&D Employees in the Pharmaceutical Industry A33	n/a
		R&D Funding A34	10 k Yuan
		No. of University Students Enrolled A35	10 k
		No. of Postgraduate Students Enrolled A36	10 k
Innovative Environment B	Economic Environment B1	Total Import and Export B11	100 M US dollar
		Actual Utilization of Foreign Capital B12	100 M US dollar
		GDP per capita B13	Yuan
	Political Environment B2	Government Fixed Asset Investment B21	100 M Yuan
		Government Expenditure on Research and Experimental Development B22	10 k Yuan
	Eco-physical Environment B3	Greenery Coverage in Urban Area B31	10 k Hectare
		Highway Mileage B32	km
		Railway Mileage B33	km
		Internet Penetration RateB34	(%)
		Mobile Phone Penetration Rate B35	(%)
	Cultural Environment B4	No. of Public Libraries B41	n/a
		No. of books in Public Libraries B42	10 k
		Turnover in Technology Market B43	100 M Yuan

Health of innovative subject refers to the ability of maintaining their own operational health, we borrow the VOR model in Costanza, Norton, and Haskell (1992) [6] as secondary indicators, whereas V, O and R are vitality, organizational force and resilience, respectively. Vitality of innovative subject measures the ability of adopting new technologies, new equipment, and new methods to provide new products and services for the society through planning, organization, coordination and other management functions, transforming innovative achievements into economic benefits. Health of the organizational structure can be measured by the quantity and stability of innovative subject. For the evaluation in resilience, we use the quantity of specialists, fundings and future researchers.

The innovative environment is the sum of the natural and social relations to innovation, innovative subject and the innovation environment reach a stable state through the input and output of flows of knowledge, technology, talent and capital, forming an innovative ecological rainforest. We divide the innovative environment into four secondary indicators: economic environment (aggregate economic condition in this area), political environment (government fiscal support), eco-physical environment (fundamental infrastructures) and cultural environment (public cultural resources and technology transaction).

The establishment of the tertiary indicators is based on the literatures of the healthiness of innovation ecological rainforest, drawing on the studies of He and Zhou (2018) [21], Liu, Zhang and Deng (2019) [11], Li and Zhang (2019) [22], Li (2019) [23], Miao and Huang (2008) [24], Zhang and Lyu (2017) [25].

### Entropy-TOPSIS model

TOPSIS method was firstly proposed by C.L. Hwang and K. Yoon in 1981 as a ranking method to approximate the ideal solution, and it has been gradually applied to the field of comprehensive evaluation in recent years. The core idea of TOPSIS method is to define the distance between the optimal solution and the worst solution to the decision problem, and finally to calculate the relative fit of each solution to the ideal solution, with corresponding rankings.

The first advantage of TOPSIS is that there is no strict restriction on the type and number of indicators, data distribution and sample size. Secondly, it can make full use of the original data, so that we can have relatively small information loss. Other similar multi-criteria decision analysis methods are AHP hierarchical analysis, DEA data envelopment analysis, etc. Analytic Hierarchy Process (AHP) is also a multi-criteria decision analysis method, which requires two relative comparisons of indicators when constructing judgment matrices. But when the number of indicators is large, it is difficult to judge that which indicators are important. In addition, the judgement is determined by researcher’s subjective choice, making results not convincing very well. Data envelopment analysis (DEA) is for multi-indicator input and output evaluation, which uses mathematical planning model to calculate the relative efficiency among comparative decision-making units (DMUs) and then constructs the evaluation. The selection of input and output terms in DEA method has a decisive influence on the efficiency evaluation results. However, the index system in this paper is constructed from two aspects of innovation subject and innovation environment, data envelopment method like DEA cannot fit this index system well.

We use the concept of entropy to determine the weight of each indicator, according to the degree of the variability. Compared with those subjective assignment methods, like direct weight, it is more accurate and objective, and can better explain the obtained results Shi et al. (2020) [26] suggest that a smaller entropy value of an indicator means more information contained and greater the role it plays in the evaluation, therefore this indicator has larger weight in the system. Further, we use entropy weighted TOPSIS method in Hwang and Yoon (1981) [27] to assess the health of innovative ecological rainforest in pharmaceutical industry, through the following steps:

Step 1. Constructing an assessment and judgement matrix.

To find the optimal evaluation objects and obtain their rankings, we build a *m*×*n* matrix containing *m* assessment objects and *n* assessment indicators. Since the number of selected indicators is large and there are great differences in dimensions and magnitude attributes among indicators, this paper standardizes the data via the Min-Max standardization method.

Step 2. Determining the weight for indicators.

We choose the information entropy weight method to determine the weight of the indicator, the information entropy *e*_*j*_ is obtained through the normalization of the indicator, whereas *f*_*ij*_ is the weight of *j*^*th*^ indicator in *i*^*th*^ year:

$$e_{j}=-\frac{1}{\ln\;n}\sum_{j=1}^{n}\left(f_{ij}\times ln\;f_{ij}\right)$$ (1)

The weight of *j*^*th*^ indicator:

$$w_{j}=\frac{1-e_{j}}{\sum_{j=1}^{m}\left(1-e_{j}\right)}$$ (2)

and $\sum_{j=1}^{m}w_{j}=1$

Step 3. Constructing the TOPSIS model.

1. Standardize original matrix and add weight to the matrix, obtaining the weighted matrix *P*.
2. Generate the best solution $P_{j}^{+}$ and the worst solution $P_{j}^{-}$ for the assessment object.
3. Calculate the best and worst solutions’ Euclidean Distance to each assessment object, $D_{i}^{+}$ and $D_{i}^{-}$; the larger $D_{i}^{+}$ and $D_{i}^{-}$ are, the further that the assessment object is from the positive ideal solution (best solution) and negative ideal solution (worst solution), respectively.
4. Calculate *M*_*i*_. Innovative ecological rainforest in pharmaceutical industry is healthier with a larger *M*_*i*_, 0≤*M*_*i*_≤1:
$$M_{i}=\frac{D_{i}^{-}}{D_{i}^{+}+D_{i}^{-}}$$ (3)
5. Finally, the health of assessment object is indicated by *oH*_*i*_ as in Table 2, where *A* is set by 100.

$$oH_{i}=A\times M_{i}$$ (4)

**Table 2:** Health of innovative ecological rainforest in pharmaceutical industry.

Health Level	Health Condition	*oH*_*i*_
Ⅰ	Weak	*oH*_*i*_<20
Ⅱ	Unhealthy	20≤*oH*_*i*_<40
Ⅲ	Critical	40≤*oH*_*i*_<60
Ⅳ	Sub-healthy	60≤*oH*_*i*_<80
Ⅴ	Healthy	80≤*oH*_*i*_

Step 4. Obstacle factor diagnosis

The pharmaceutical innovation ecological rainforest health assessment index system contains two dimensions, and each dimension contains several indicators. Furthermore, there is variability in the real impact of each dimension and indicator on the health of pharmaceutical innovation ecological rainforest. In order to further investigate the impact, we introduce the Obstacle factor diagnosis model to find out the key constraints. We follow Kuang et al. (2018) [28] to include factor contribution, indicator deviation and degree of obstacle in the model:

$$M_{i}=w_{j}\times P_{ij}$$ (5)

$$I_{i}=1-R_{i}$$ (6)

$$Q_{j}=\frac{I_{j}\times F_{j}}{\sum_{j=1}^{n}\left(I_{j}\times F_{j}\right)}\times 100\%$$ (7)

$$U_{j}=\sum Q_{ij}$$ (8)

whereas *F*_*j*_ is deviation of *j*^*th*^ indicator, *w*_*j*_ is the weight of *j*^*th*^ indicator, *P*_*ij*_ is the weight of *j*^*th*^ indicator under *i*^*th*^ level, *I*_*j*_ is factor contribution, *R*_*j*_ is the standardized value for the original matrix, *Q*_*j*_ is the degree of obstacle from *j*^*th*^ indicator to the construction of pharmaceutical innovation ecological rainforest, *U*_*j*_ is degree of obstacle from each level of indicator to the construction of pharmaceutical innovation ecological rainforest.

### Data

The corresponding data are captured from the “China High Technology Industry Statistical Yearbook”, “Zhejiang Province Statistical Yearbook”, “Zhejiang Province Internet Development Report”, and “China Statistical Yearbook”.

### Empirical results

#### Health assessment index system of innovative ecological rainforest in pharmaceutical industry

**Fig 1:**
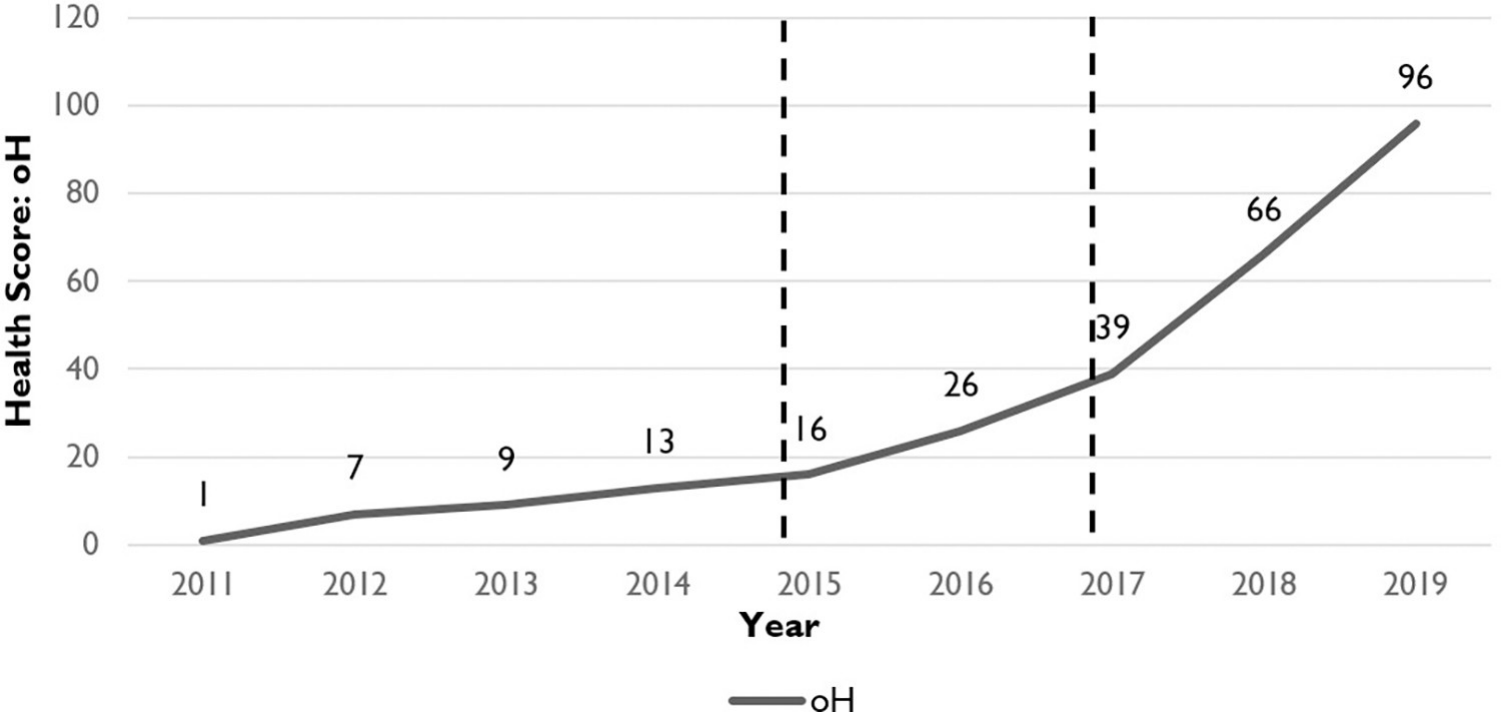
Health of pharmaceutical innovative ecological rainforest in Zhejiang Province: 2011–2019.

Table 3 shows the entropy value and weight of all indicators, and Table 4 shows the outcome of health assessment for pharmaceutical innovative ecological rainforest in Zhejiang Province. From 2011 to 2019, clearly in Fig 1, there was a continuous upward trend in the health level of pharmaceutical innovative ecological rainforest in Zhejiang Province, and the health score increased from 1 (weak) in 2011 to 96 (healthy) in 2019.

**Table 3:** Entropy value and weight of indicators in pharmaceutical innovative ecological rainforest in Zhejiang Province.

Primary indicator	Secondary indicator	Tertiary indicator	Entropy	Weight
A	A1	A11	0.1117	0.0311
		A12	0.2308	0.0643
		A13	0.1283	0.0358
		A14	0.1175	0.0328
	A2	A21	0.0718	0.0200
		A22	0.0899	0.0251
		A23	0.1425	0.0397
		A24	0.1346	0.0375
	A3	A31	0.0806	0.0225
		A32	0.0736	0.0205
		A33	0.0996	0.0278
		A34	0.1232	0.0343
		A35	0.1094	0.0305
		A36	0.1680	0.0468
B	B1	B11	0.1919	0.0535
		B12	0.1075	0.0299
		B13	0.1402	0.0391
	B2	B21	0.1050	0.0293
		B22	0.0889	0.0248
	B3	B31	0.0941	0.0262
		B32	0.0980	0.0273
		B33	0.1435	0.0400
		B34	0.1457	0.0406
		B35	0.0818	0.0228
	B4	B41	0.1892	0.0527
		B42	0.1317	0.0367
		B43	0.3893	0.1085

**Table 4:** 2011–2019 health of pharmaceutical innovative ecological rainforest in Zhejiang Province.

Year	Positive ideal solution D^+^	Negative ideal solution D^-^	Relative closeness M	oH	Health level
2011	0.0855	0.0011	1.2984	1	Ⅰ
2012	0.0830	0.0058	6.5557	7	Ⅰ
2013	0.0820	0.0081	8.9514	9	Ⅰ
2014	0.0800	0.0118	12.8315	13	Ⅰ
2015	0.0781	0.0152	16.3051	16	Ⅰ
2016	0.0675	0.0240	26.2138	26	Ⅱ
2017	0.0547	0.0349	38.9981	39	Ⅱ
2018	0.0292	0.0578	66.4721	66	Ⅲ
2019	0.0030	0.0855	96.4907	96	Ⅴ

According to the health score and health level, the pharmaceutical innovative rainforest ecology in Zhejiang Province can be divided into three stages:

Stagnation period (2011–2015): At this stage, the health of Zhejiang pharmaceutical innovative ecological rainforest was significantly affected by policy and market factors. On the one hand, there were substantial adjustments in national pharmaceutical policies during this period, State Council’s ’Twelfth Five-Year Plan for National Drug Safety’ put forward a statement of consistency evaluation of generic drugs for the first time; in 2011, the Ministry of Health issued the ’Good Manufacturing Practice for Drugs (New GMP) (Revised in 2010)’, which set higher requirements for key personnel from the aspects of academic qualifications, technical titles, work experience, etc., pushing enterprises to establish a management system with higher quality. On the other hand, the international environment was complex, the export-oriented pharmaceutical industry in Zhejiang Province was in a ’cold winter’, because of the European Debt crisis.

Recovery period (2015–2017): The health score increased from 16 (weak) in 2015 to 39 (unhealthy) in 2017. During this period, Zhejiang Province issued the policy of ’Implementation Opinions on Accelerating the Innovative Development of the Pharmaceutical Industry’ and many industry clusters started, including Hangzhou Future Sci-tech City, Yuhang Biomedical High-Tech Industrial Area and Hangzhou Biopharma Town, with the development of artificial intelligence and digital economy.

Development period (2017–2019): The health of the innovative ecological rainforest was significantly improved, and the health score increased from 39 (unhealthy) in 2017 to 96 (healthy) in 2019. At this stage, many policies in Zhejiang Province were further strengthened and biomedicine had been included in the key areas of ’Zhejiang Manufacturing Excellence’. As of November 2020, there were 50 pharmaceutical enterprises listed in Shanghai and Shenzhen stock markets, ranking at the first place of all provinces in China. The concept of digital was introduced to pharmaceutical industry, establishing new modes of internet hospital and third-party diagnosis.

### Health condition in innovative subject

**Fig 2:**
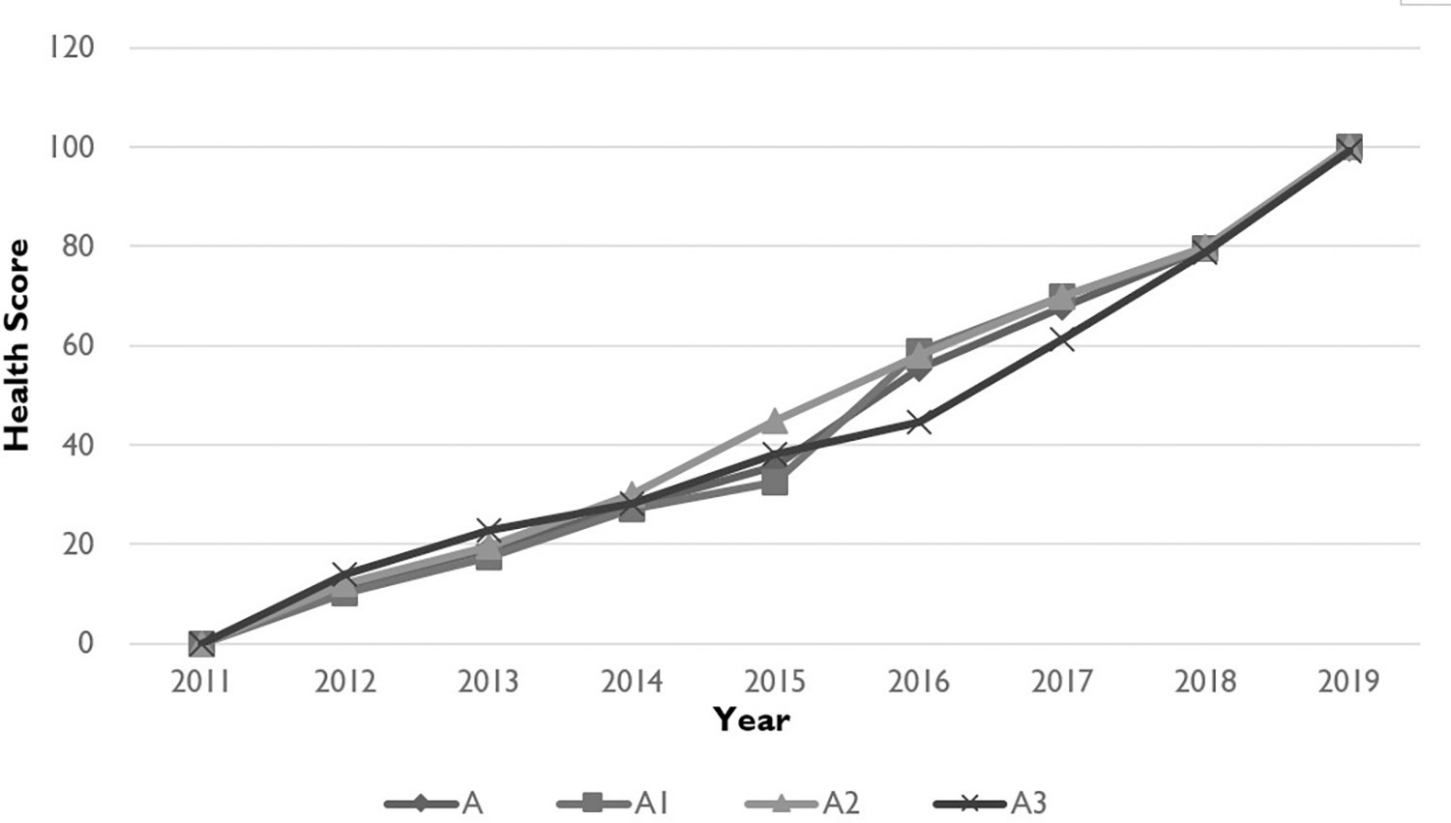
Health of innovative subject in pharmaceutical innovative ecological rainforest in Zhejiang Province: 2011–2019.

As shown in Fig 2, from 2011 to 2019, the health of innovative subjects in the pharmaceutical industry in Zhejiang Province was improved, from the lowest level I (weak)to the level V (healthy). We could see a sharp improvement in three aspects of innovative subject: vitality (A1), organizational structure (A2) and resilience (A3), since the health scores of them reached 100 in 2019.

### Health condition in innovative environment

**Fig 3:**
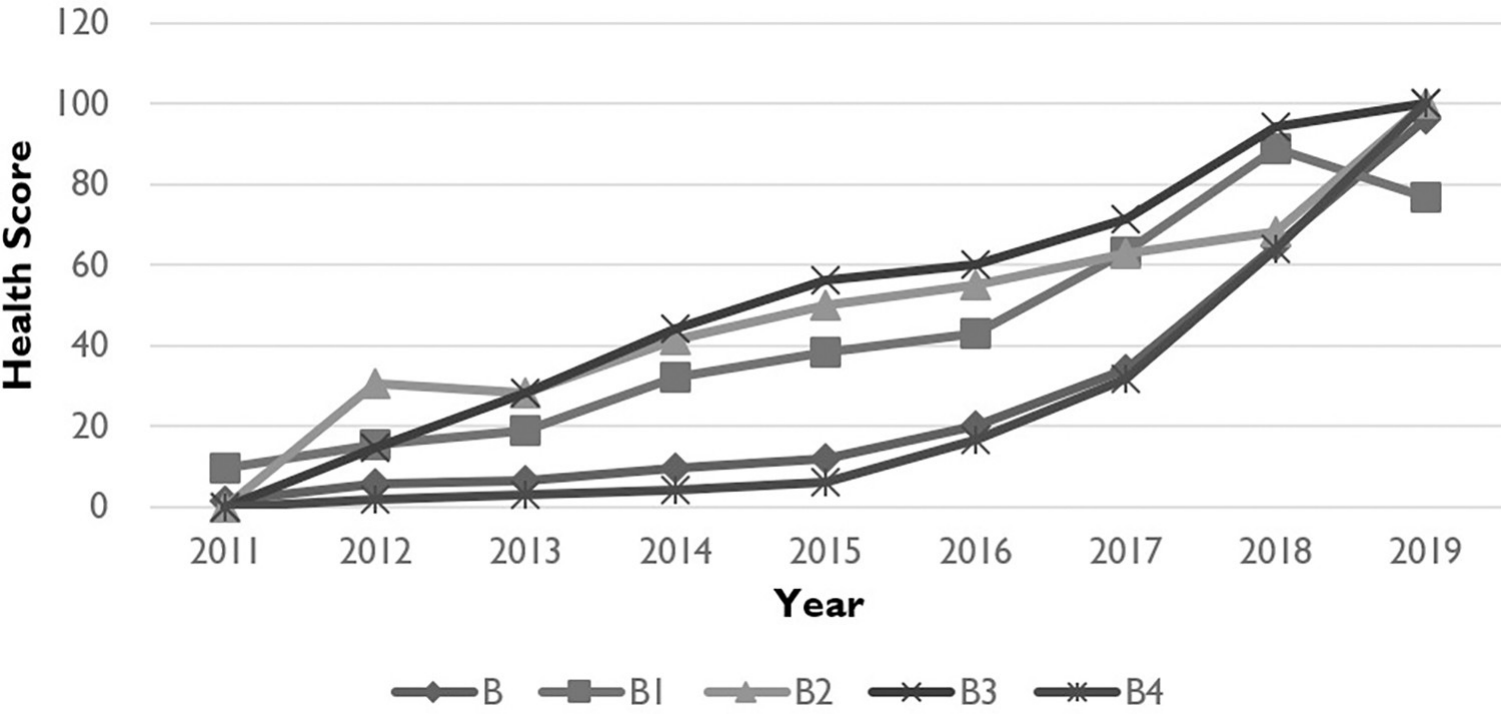
Health of innovative environment in pharmaceutical innovative ecological rainforest in Zhejiang Province: 2011–2019.

Fig 3 shows that the health of the innovative environment of pharmaceutical industry in Zhejiang Province has been greatly improved in recent years. The year of 2016 is an important breakpoint, because it stayed at weak level before 2016 but the health score increased from 20 in 2016 to 96 in 2019. There was a steady development in Zhejiang Province, in terms of economic environment (B1), political environment (B2) and ecological physical environment (B3). In 2019, the health score of both political environment and eco-physical environment raise to 100, but the external economic condition deteriorated due to the rise of the global trade protection and Sino-US trade frictions, so there was a slight decline in health score of the economic environment. Regarding to the cultural environment, the year of 2017 is an important inflection point, because of the rapid development in the scale of transaction in technology market: the transaction volume of the technology market in Zhejiang Province was 7.19 billion yuan in 2011, and it reached 88.801 billion yuan in 2019.

### Obstacle analysis

**Fig 4:**
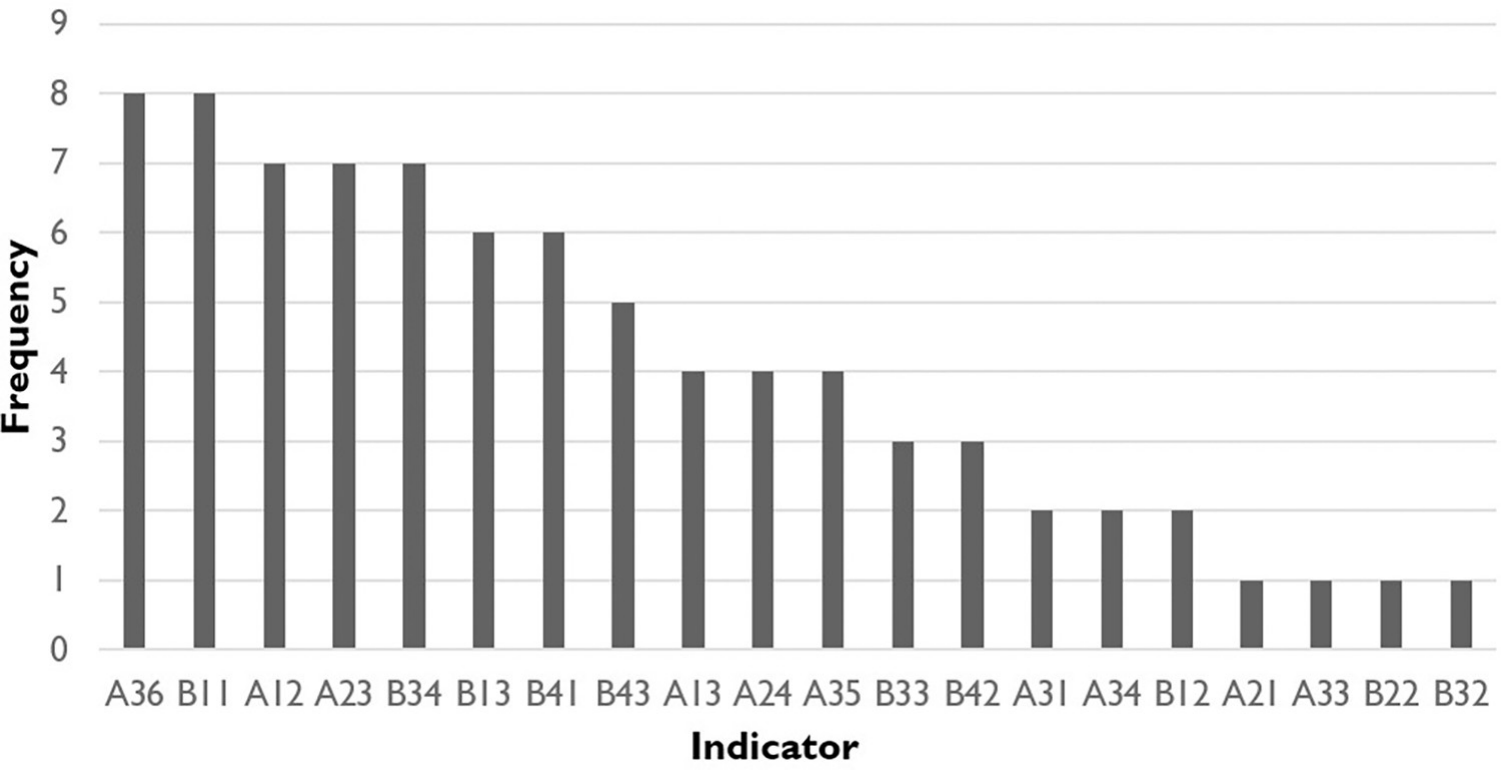
Frequency of top 10 indicators of obstacles to pharmaceutical innovative ecological rainforest in Zhejiang Province: 2011–2019.

As shown in Table 5 and Fig 4, the frequency of indicators in innovation environment is slightly higher than those in innovation subjects, with frequencies of 42 and 40 respectively, indicating that the innovation environment is an important obstacle factor hindering the development of the pharmaceutical innovative rainforest ecology in Zhejiang Province. The resilience of innovative subject is ranked at the first place among all obstacle factors, with frequency of 17, mainly because of the weakness in the number of postgraduate students (A36) and the number of pharmaceutical enterprises (A31). Economics environment is ranked at the second place, since the uncertainty of world economy increases the risk of the healthy development of the pharmaceutical industry in Zhejiang Province. The cultural environment with the frequency of 14 is ranked at the third place due to the slow development of the technology market in Zhejiang Province from 2011 to 2016, which causes the insufficient flow between various elements in the innovative ecology rainforest, thus affecting the health of the pharmaceutical innovation ecological rainforest.

**Table 5:** Top 10 indicators of obstacles to pharmaceutical innovation ecological rainforest in Zhejiang Province.

Order	2011	2012	2013	2014	2015	2016	2017	2018	2019
1	0.08	0.08	0.07	0.08	0.08	0.11	0.17	0.28	0.93
	B41	B41	B41	B41	A12	B43	B43	B43	B12
2	0.07	0.07	0.07	0.08	0.08	0.10	0.08	0.07	0.07
	B11	B11	A12	A12	B11	B11	B11	A13	A31
3	0.06	0.06	0.07	0.07	0.07	0.07	0.06	0.06	-^a^
	A12	A12	B11	B11	A36	A36	A36	A36	
4	0.05	0.05	0.06	0.06	0.06	0.06	0.05	0.05	-
	A36	A36	A36	A36	B43	B34	A13	B22	
5	0.05	0.05	0.05	0.05	0.06	0.06	0.05	0.05	-
	B34	B34	B34	B34	B41	A12	B41	A23	
6	0.04	0.05	0.04	0.05	0.05	0.05	0.05	0.05	-
	B33	B33	B13	A23	B34	A13	B34	A35	
7	0.04	0.04	0.04	0.05	0.05	0.05	0.04	0.04	-
	A24	B13	A24	B13	A23	A23	A23	A21	
8	0.04	0.04	0.04	0.05	0.05	0.04	0.04	0.04	-
	A35	A24	B33	B43	B13	B13	A12	A33	
9	0.04	0.04	0.04	0.04	0.04	0.04	0.04	0.04	-
	B13	A23	A23	A24	A13	A34	A34	A31	
10	0.04	0.04	0.04	0.04	0.04	0.04	0.04	0.03	-
	B32	A35	B12	B42	B42	B42	A35	B11	

^a^ In 2019, obstacle values are all zero, except for B12 and A31

### Discussion of findings and suggestions

Regarding to health of the innovation subject in the pharmaceutical industry, we can see a great improvement from 2011 to 2019. In terms of the vitality of the innovation subject, there was a significant growth in business revenue, total profit and new product sales revenue. The number of patent applications in the pharmaceutical industry increased from 958 in 2011 to 2939 in 2019. From 2011 to 2019, the health of the innovation subject of the pharmaceutical industry in Zhejiang Province has been improved. In terms of the vitality of the innovation subject, there was a significant growth in business revenue, total profit and new product sales revenue. The number of patent applications in the pharmaceutical industry increased from 958 in 2011 to 2,939 in 2019, showing increasing innovation capacity. In terms of organizational structure, the number of R&D institutions in the pharmaceutical industry in Zhejiang Province increased from 240 to 336, with a significant increase in basic innovation capacity, providing a solid base for innovation. The year-end balance of deposits (local and foreign currency) of financial institutions and the number of law firms in Zhejiang Province increased at the same time, providing financial and legal support for the pharmaceutical industry. In terms of resilience, the number of pharmaceutical industry enterprises, the average number of employed personnel and the number of R&D personnel in Zhejiang Province all grew significantly. In addition, the number of university students and postgraduates kept rising, from whom we saw a strong innovation potential in the future. Finally, the investment in R&D funding raise from 407 million yuan in 2011 to 1.12 billion yuan in 2019.

Regarding to health of the innovation environment in the pharmaceutical industry, e can see a great improvement from 2011 to 2019 as well. In terms of economic environment, Zhejiang Province has been in a stage of rapid development, but due to the rise of global trade protection and trade friction between China and US, economic environment has deteriorated slightly with fluctuation in the trade and the actual utilization of foreign capital. In terms of political environment, the development of the pharmaceutical industry is inseparable from the guidance and support from the government. The investment in fixed assets by the Zhejiang Provincial Government increased from 1407 billion yuan in 2011 to 3427 billion yuan in 2019. Research and experimental development expenditure was also in a growth state during the period, except for a small decline in 2013. In terms of ecological and physical environment, Zhejiang Province is famous for its digital economy, with high internet penetration rate and mobile phone penetration rate, reaching 80.9% and 152.3% respectively. In terms of cultural environment, the development of the private economy has achieved the inclusive and open cultural character of Zhejiang Province, which is beneficial for the innovation and creation.

### Suggestions

Based on the outcomes of the health assessment, we suggest following measures to build a pharmaceutical innovative ecological rainforest and boost the high-quality development of the pharmaceutical industry in Zhejiang Province:

### Creating a pharmaceutical industry chain innovation pattern and deploying a high-level service chain

In general, we should deepen the implementation of pharmaceutical innovation-driven development strategy, promote the formation of a new pattern of innovation and entrepreneurship development with the synergy of government, industry, academia and research, the integration of large and small enterprises, so that elements in the rainforest of innovation are able to flow freely and smoothly. Since the in-depth cooperation among universities, research institutes and pharmaceutical enterprises could gather high-end innovation resources, the supply for the technology innovation will be strengthened, and research outcomes could further support the technology development, technology consulting, technology services, technology training to the pharmaceutical innovation and entrepreneurship, thickening and broadening the foundation of pharmaceutical innovation rainforest.

### Improving the investment chain of pharmaceutical innovation and broadening the investment and financing channels

The financial support is important for the start-up and later development of the innovation rainforest. We suggest that the government fiscal support, as subsidiary and tax reduction, may target the innovation by the pharmaceutical enterprises, especially those focus on the high-end technology. Furthermore, financial institutes are encouraged to provide various credit product, ranging from physical assets credit to knowledge assets credit, under the background that the law of intellectual property protection has taken a great step forward in recent years.

### Attracting biomedical talents and building a global talent pool

One of the weaknesses in the Zhejiang pharmaceutical industry, compared with other province nearby, is the lack of high-end talent. Thus, the local government and enterprise may work together, improving the long-term talent service mechanism and creating a full life-cycle talent service system. Enterprise may further cooperate with high education and research institutes to train the talent from the very beginning, with long-term and effective practical training bases.

### Improving the government services and creating an inclusive and open soft environment

Zhejiang Province has a mature and complete digital system, it can be seen in government, business, education and other daily life. We should take full use of the digital economy and digital governing to fight against with the counterfeit production and sale. In addition, a social culture of inclusive and open is important for the innovation and creation as well. Hight tolerance of failure would actively encourage more enterprise to do the high risk and high investment innovation.

## Conclusion

This paper constructs a health assessment index system for the pharmaceutical innovative ecological rainforest in Zhejiang Province, including seven secondary indicators of the vitality, organizational structure and resilience for the innovation subject, economic environment, political environment, the eco-physical environment and the cultural environment for innovative environment. Through the entropy weighted TOPSIS method and the obstacle factor diagnosis model, we evaluate the health of the innovative ecological rainforest in the pharmaceutical industry of Zhejiang Province from 2011 to 2019.

Overall, the development of the pharmaceutical innovative ecological rainforest in Zhejiang Province can be roughly divided into three stages: a stagnation period with a low level of health for a long time; a recovery period during which the health was steadily improved, and industrial clusters were formed; a development period that the health level of the pharmaceutical innovative ecological rainforest in Zhejiang Province reached the historical high, with a great improvement in the innovation vitality. We could see a relatively balanced development in the health of the innovative subject and the innovative environment from 2011 to 2019, but health of the pharmaceutical innovation ecological rainforest in Zhejiang Province has been greatly improved in vitality, organizational structure and resilience. In terms of obstacle factors, the frequency of innovative environment is slightly higher than the frequency of innovative subject, and the resilience in innovative subject is the key factor that hindering the development of the pharmaceutical innovative ecological rainforest in Zhejiang Province, followed by the economic environment and cultural environment.

To further improve the ecological rainforest of pharmaceutical innovation and promote the high-quality development of the pharmaceutical industry, we propose four countermeasures, including "deploying high-level service chains, broadening investment and financing channels for enterprises, attracting talents, and creating an inclusive and open soft environment".

There are some limitations of this study: First, the conceptual understanding needs to be deepened. The concept of innovative ecological rainforest is in its embryonic stage, and pharmaceutical innovative ecological rainforest is a complex system involving multiple disciplines and fields; it means that the understanding of innovative ecological rainforest is inevitably subjective, and the depth and breadth of its understanding still needs further exploration. Second, the index system needs to be optimized. Due to the availability of data, the selection of indicators is not innovative enough to fully display the connotation and characteristics of innovative ecological rainforest. Third, implication is not global but local. This paper only analyses the health status of the pharmaceutical innovation ecological rainforest in Zhejiang Province from 2010–2019, without extension to the national scale or worldwide. Finally, although this paper uses the obstacle factor diagnostic model to determine the factors that hindering the health of the pharmaceutical innovation ecological rainforest in Zhejiang Province, it indeed does not study the impact mechanism. All these can be improved in the future works.

## Supporting information

S1 Data (XLSX)
